# Analysis of Expression and Single Nucleotide Polymorphisms of* INHA* Gene Associated with Reproductive Traits in Chickens

**DOI:** 10.1155/2019/8572837

**Published:** 2019-08-08

**Authors:** Zhifu Cui, Lingbin Liu, Xiaoling Zhao, Jinshan Ran, Yan Wang, Huadong Yin, Diyan Li, Qing Zhu

**Affiliations:** Farm Animal Genetic Resources Exploration and Innovation Key Laboratory of Sichuan Province, Sichuan Agricultural University, Chengdu 611130, China

## Abstract

Inhibin *α* (*INHA*) is a candidate gene controlling ovulation in poultry. As the functional center of inhibin,* INHA *is a molecular marker associated with egg-laying performance. The objective of the current study was to analyze the expression differences of* INHA* in reproductive system and single nucleotide polymorphisms (SNPs) associations with reproductive traits in chickens. A total of 260 LuHua chickens (barred-feather chicken) were adopted. Twelve SNPs were detected in* INHA* gene. Among the exonic SNPs, three (g. 22177991A>G, g. 22178249G>C, and g. 22178414G>A) were missense mutations, resulting in the amino acid substitutions Val→Ala, Ala→Gly, and Ala→Gly, respectively. Four SNPs in the 3＇ untranslated region of* INHA* were predicted to either disturb or create microRNA-target interactions. Five SNPs (g. 22176870T>C, g. 22177100T>C, g. 22177149T>C, g. 22177991A>G, and g. 22178975G>A) were significantly associated with the number of eggs at 300 d of age (EN) (*P *< 0.05). Birds carrying GA genotype exhibited more EN than those with AA genotype (*P *< 0.01). In addition, quantitative real-time PCR revealed that* INHA* is mainly expressed in follicles on d 300 in chickens. Firstly,* INHA *expression increased and then decreased. The highest* INHA* mRNA abundance was found in the fifth largest preovulatory follicle (F5) (*P *< 0.01). In the prehierarchical follicles,* INHA* mRNA expression increased dramatically in small yellow follicles (SYF) (*P *< 0.01). Western blotting analysis showed that the INHA protein expression profile in the follicle was similar to its mRNA counterpart with greater expression in F5 and SYF follicles and lowest expression in F1 follicles (*P* < 0.05). These results suggest that* INHA *is a potential candidate gene improving reproductive traits in chickens.

## 1. Introduction

Inhibin, a member of the transforming growth factor-*β* (TGF-*β*) superfamily, plays an important role in modulating the reproductive axis and affects all reproductive events [1]. It is a gonadal glycoprotein hormone and is principally produced by the granulosa cells of ovarian follicles in females and by the sertoli cells of the testes in males [2]. Inhibin comprises multiple disulfide-linked dimers that shares a common *α*-subunit (encoded by* INHA *gene) and differs in *β*-subunit; when the *α*-subunit binds to *β*A-subunit, inhibin A (*αβ*A) is formed, and when it binds to the *β*B-subunit, inhibin B (*αβ*B) is formed. Both inhibin A and inhibin B have the ability to specifically suppress follicle-stimulating hormone (FSH) secretion in pituitary cells without affecting LH secretion [3–7]. In addition to its endocrine function, inhibin also shown to exert a variety of autocrine/paracrine in mammalian species. Many studies indicated a local role for these factors in modulating the growth of small follicles by regulating cell proliferation as well as expression of the inhibin subunits and gonadotropin receptors [8, 9].

Inhibin A is produced and secreted by the granulosa layer of the large pre-ovulatory follicle [10]. Immunizations against the* INHA* have resulted in increased ovulation in sheep, pigs, chickens, mice, and cows [11–15]. Therefore, the* INHA* is thought to be the functional center of inhibin and a potential candidate gene increasing the ovulation rate in poultry. In a murine knockout model, the* INHA* was found to play a tumor suppressive role in gonadal tissue after gonadectomy for the adrenal cortex, with 99% of* INHA* mice developing adrenocortical steroid-secreting carcinoma after gonadectomy [16, 17]. Pathways involved in this effect include the differentiation into granulosa cell-like cells with the expression of fetal or gonadal markers such as* LHR*,* FSHR,* and* Cyp17a1* [18]. Recently, Li et al. found that* INHA* gene polymorphisms are significantly associated with the presence of follicular cysts in Large White sows [19]. In poultry, Chen et al. reported that the downregulation of* INHA* expression has an antagonistic effect on granulosa cell apoptosis [20]. Although many studies have shown that inhibin was a critical regulator of gonadal function, whether its expression level in the developing follicles or polymorphisms are associated with reproductive traits in chicken is still unknown.

In characterizing candidate genes, it is important to detect single nucleotide polymorphisms (SNPs) and analyze the associations between these SNPs and reproductive traits, together with assessing the role of gene expression. Therefore, the aim of this study was to characterize the role of the chicken* INHA* gene in determining chicken reproductive traits through SNP association and expression analyses.

## 2. Materials and Methods

### 2.1. Chicken Populations

The LuHua chicken (LH) is a local breed in Shandong province, P. R. China. It was included in the Key Poultry Breeds Requiring Protection in 2002. A total of 260 birds after hatching were raised in cages with the same housing conditions and diets. Their performances were measured in Poultry Breeding Farm of Sichuan Agricultural University (Ya'an, China). Blood was collected, and genomic DNA was isolated using a standard phenol/chloroform method. The DNA purity and concentration were measured with a Nucleic Acid Protein Analyzer (Thermo Scientific, USA). Based on the original concentration, samples were diluted to the final concentration of 100 ng/*μ*L with Tris-EDTA buffer. All DNA samples were stored at -20°C until use.

Six reproductive traits were measured: body weight at first egg (BWAFE), first egg weight (FEW), age at first egg (AFE), total egg number at 300 days of age (EN), body weight at 300 days of age (BWTA), and egg weight at 300 days of age (EWTA). Traits determination was conducted in accordance with the Committee on Experimental Animal Management of Sichuan Agricultural University and carried out strictly according to the Regulations for the Administration of Affairs Concerning Experimental Animals of the State Council of China.

### 2.2. Sequencing of the INHA Gene

Primers for amplifying and sequencing the chicken* INHA* gene (Table 1) were designed with Primer Premier 5.0 software based on the complete DNA sequences of* Gallus gallus INHA* genes (ENSGALG00000054770). A DNA pool containing 100 ng DNA from each of the 60 least closely related chickens was constructed. PCR was carried out using a Gene Amp PCR System 9700 (Bio-Rad, Hercules, CA, USA) thermal cycler in a final volume of 25 *μ*L containing 8.6 *μ*L distilled H_2_O, 15 *μ*L 2×Taq PCR Master Mix (including Mg^2+^, dNTPs, and Taq DNA polymerase; Beijing Tian Wei Biology Technique Corporation, Beijing, China), 0.3 *μ*L each primer (10 nmol/L), and 0.8 *μ*L chicken genomic DNA template. PCR was performed with the following cycling conditions: denaturation at 94°C for 5 min; 35 cycles of 94°C for 30 s, 56-60°Cfor 35 s (Table 1), and 72°C for 40 s, followed by a final extension at 72°C for 5 min. PCR products were purified and sequenced via an ABI 377 DNA sequencer (Shanghai Sangon Biological Engineering Technology, Shanghai, China). All sequences were edited, assembled, and aligned with DNASTAR and Codon Code Aligner software (http://www.codoncode.com/aligner). SNPs were identified by the presence of multiple peaks at the same base by direct sequencing.

### 2.3. Genotypes Identification

Based on the DNA pools sequence results, single nucleotide polymorphisms were found in the amplification products of five primer pairs P1, P2, P3, P4, and P5. Further polymorphism genotyping was carried out one by one using DNA samples of 260 chickens. PCR and amplified products were carried out as describe as above.

### 2.4. Statistical Analyses

SAS 9.4 (Statistical Analysis Systems Institute Inc., Cary, NC, USA) was used to determine the relationship between genotypes and reproductive traits in chicken. The model used was: Y_i_ = *μ* + G_i_ + e_i_, where Y_i_ is a reproductive trait measured in the chickens, *μ* is the population mean of the trait, G_i_ is a fixed effect genotype, and e_i_ is the residual error. Significance was determined using Duncan's multiple range tests. Pearson's chi-square test was used to assess the Hardy-Weinberg equilibrium of the four SNPs discovered in the present study. The linkage disequilibrium (LD), D＇ and r^2^ values of the SNPs were estimated by Haploview [21]. The polymorphism information content (PIC) was determined following a previous method [22]. In addition, haplotypes were constructed using the PHASE program v. 2.0. Haplotypes were analyzed using the model for the single marker association test by considering birds with 0, 1, or 2 copies of the haplotype in question. The PROC REG procedure of SAS (version 9.4, SAS Institute Inc.) was used to perform the analysis. Values were significant at* P* < 0.05.

### 2.5. Expression Abundances of INHA mRNA

To determine the reproductive system tissue-specific expression patterns of* INHA* mRNA, total RNA was extracted from the tissues of five chickens at 25 weeks, including the fallopian tubes (infundibulum, isthmus, and uterus), hierarchical follicles (F1, F2, F3, F4, and F5), and prehierarchical follicles [small white follicles (SWF), small yellow follicles (SYF), and large white follicles (LWF)], using TRIzol reagent (TakaRa Biotech Co. Ltd., Dalian, China) and was dissolved in RNase-free H_2_O (Tiangen Biotech Co., Ltd, Beijing, China). The integrity of the RNA was evaluated via electrophoresis on 1 % agarose gels, and the concentration and purity were analyzed with a NanoDrop 2000 by determining the absorbance ratio of 260/280 nm (Thermo Scientific, Waltham, MA, USA). The cDNA was synthesized by reverse-transcription PCR using 1 *μ*g total RNA, 2 *μ*L 5 × RT buffer, 0.5 *μ*L RT enzyme mix, 0.5 *μ*L primer mix, and 6 *μ*L nuclease-free water (Toyobo Life Science Department, Shanghai, China). The reverse transcription reaction was maintained at 37°C for 15 min, followed by incubation at 98°C for 5 min. The cDNA samples were stored at -20°C. Gene-specific primers (Fw: 5＇-ACTACTGCCACGGGAACTGT-3＇, Rv: 5＇-GGAGTAGCCACCATCAGAGG-3＇) for qRT-PCR were designed using Primer 5 software according to the coding sequence of the chicken* INHA* gene (GenBank accession no. NM_001031257). qRT-PCR was conducted via a CFX96 Real-time System (Bio-Rad, Hercules, CA, USA) with the following conditions: 98°C for 2 min; 39 cycles of 98°C for 5 s and 55.7°C for 10 s; followed by a final extension at 95°C for 10 s. Each PCR reaction contained 5 *μ*L Ssofast Evagreen supermix (Bio- Rad, Hercules, CA, USA), 1 *μ*L of cDNA (50 ng/*μ*L), 0.8 *μ*L each primer (10 *μ*M), and 2.4 *μ*L ddH_2_O to a final reaction volume of 10 *μ*L. All samples were triplicated. The housekeeping gene* β-actin* (GenBank accession no. AF047874; Fw: 5＇-GAGAAATTGTGCGTGACATCA-3＇, Rv: 5＇-CCTGAACCTCTCATTGCCA-3＇) was used for normalization of target gene expression [23]. The relative gene expression levels of* INHA* were calculated using the comparative 2^−ΔΔCT^ method [24], where ΔCt = Ct target gene - Ct housekeeping gene. Differences in* INHA* mRNA expression were examined by one-way analysis of variance (ANOVA). Data are presented as the mean ± standard error means (SEM), and significances were determined at* P *< 0.05.

### 2.6. Expression Abundances of INHA Protein

INHA protein expression levels in different follicles were detected by Western Blotting. Total protein was extracted from the tissues of five chickens at 25 weeks, including the hierarchical follicles (F1, F2, F3, F4, and F5) and prehierarchical follicles (SWF, SYF, and LWF) using a Protein Extraction Kit (BestBio Biotech Co. Ltd., Shanghai, China). The concentration and purity of the protein samples were determined using a BCA Protein Assay Kit (BestBio Biotech Co. Ltd., Shanghai, China); standard curves were drawn to calculate the protein concentration. Samples were triplicated. The *β*-actin protein was used as a reference. A total of 25 *μ*g protein was resolved by sodium dodecyl sulfate-polyacrylamide gel electrophoresis (SDS-PAGE) and transferred to polyvinylidene fluoride (PVDF) membranes. After blocking with 5 % non-fat milk in 1×Tris-buffered saline with Tween (TBST) buffer for 2 h at room temperature, membranes were incubated with rabbit anti-chicken* INHA* monoclonal antibody (Abcam, Cambridge, UK; 1:1000) and rabbit anti-chicken *β*-actin monoclonal antibody (Abcam, Cambridge, UK; 1:1000) overnight at 4°C. Blots were then washed in 1 × TBST buffer and probed with goat anti-rabbit horseradish peroxidase (HRP)-conjugated IgG secondary antibody (diluted 1:2000 in 1 × TBST; Abcam, Cambridge, UK) for 1 h at room temperature. Binding was visualized with enhanced chemiluminescence (ECL) reagent (Beyotime Institute of Biotechnology, Jiangsu, China) using a ChemiDoc XRS instrument (Bio-Rad, Hercules, CA, USA). Quantity One Software (Bio-Rad, Hercules, CA, USA) was used for densitometric analysis [25].

## 3. Results

### 3.1. Allele and Genotype Frequencies of Chicken INHA Gene SNPs

**Figure 1:**
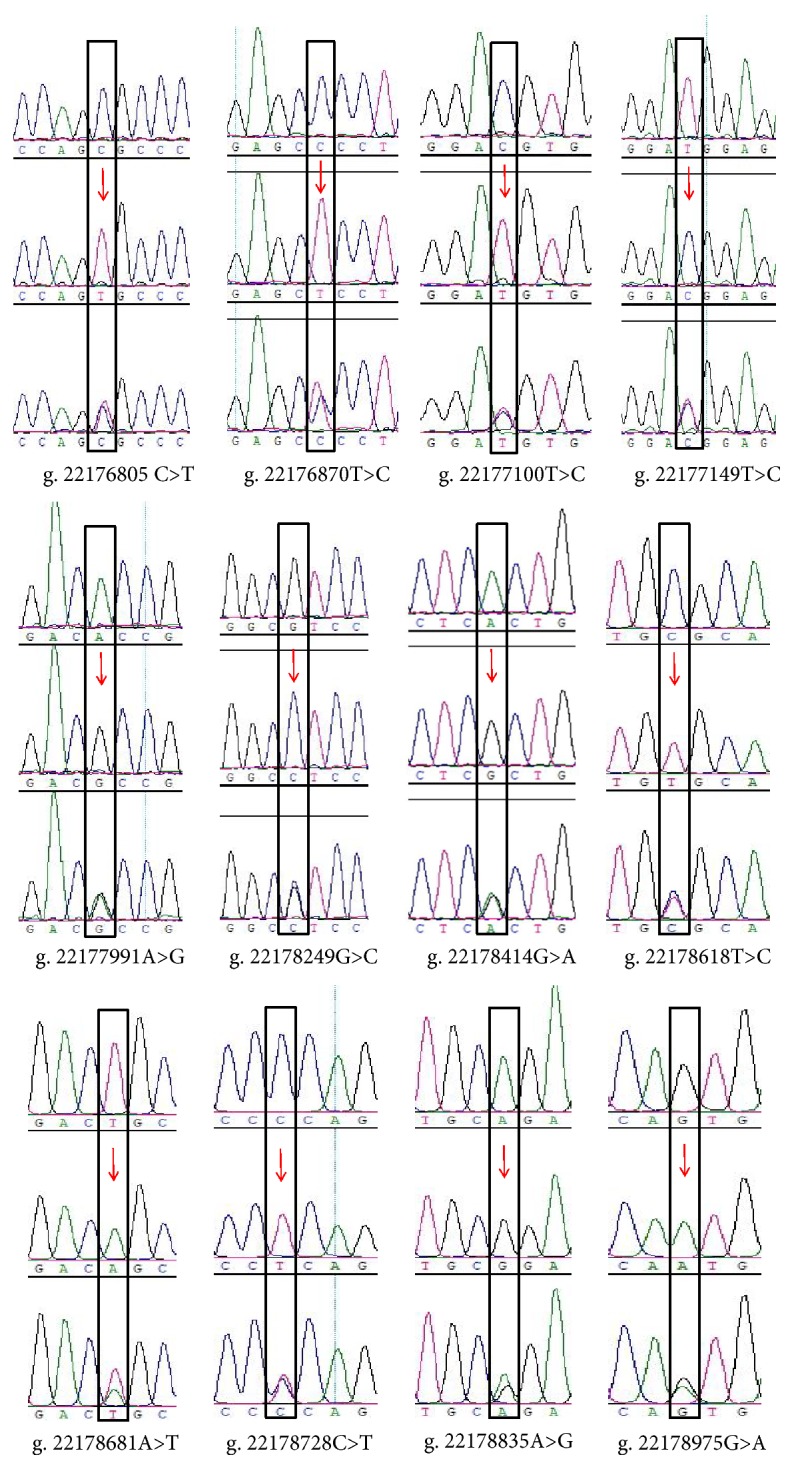
SNP sequence map of* INHA* gene. Red arrow indicates mutation site.

All exons and part of the untranslated region (UTR) of the chicken* INHA* gene were amplified and sequenced. A total of 12 SNPs were detected (Figure 1). Among them, one was located in the promoter region, three were in exon 1, three were in exon 2, and five were in the 3＇ UTR (Table 2). Among the exonic SNPs, g. 22177991 A>G, g. 22178249 G>C, and g. 22178414 G>A were missense mutations, which resulted in the amino acid substitutions Val→Ala, Ala→Gly, and Ala→Gly, respectively. SNPs in the 3＇ UTR were predicted to either disturb or create microRNA- (miRNA-) target interactions. Ten miRNA interactions weakened and four miRNA interactions strengthened (Table 3).

The genotype and allele frequencies and related genetic information for the 12 SNPs in the chicken* INHA* gene are summarized in Table 4. For all SNPs, their genotype and allele frequencies were above 5 %, which indicated that it was appropriate to conduct* INHA* gene analysis. For SNP1, the CT genotype frequency (0.6121) was higher than those of CC (0.3103) and TT (0.0776), and the allele frequency of C (0.61635) was higher than that of T (0.38365). The heterozygous genotype frequency was higher than the homozygous genotype frequencies for all 12 SNPs. The original allele frequency was higher than the mutant allele frequency for SNP1, SNP2, SNP3, SNP4, SNP7, SNP8, and SNP9, respectively, while the original allele frequency was lower than the mutant allele frequency for SNP5, SNP6, SNP10, SNP11, and SNP12. The PIC test results indicated that all SNPs could be considered intermediate polymorphisms, making them good genetic markers. All SNPs except SNP1 were in Hardy-Weinberg equilibrium (*P *> 0.05).

### 3.2. Relationships between Genotypes and Reproductive Traits

The results of the association analyses showed that g. 22176870T>C, g. 22177100T>C, g. 22177149T>C, g. 22177991A>G, and g. 22178975G>A were significantly associated with EN (*P *< 0.05, Table 5). No significant associations were found between other SNPs and the reproductive traits. Therefore, subsequent genotype association analyses were performed for g. 22176870T>C, g. 22177100T>C, g. 22177149T>C, g. 22177991A>G, and g. 22178975G>A (Table 6). The results showed that genotypes at the g. 22176870T>C, g. 22177100T>C, g. 22177149T>C SNPs were significantly associated with EN (*P* < 0.05). Birds with the TC genotype at these SNPs had significantly higher ENs than those with the CC genotype. At the* INHA* exon 2 SNP g. 22177991A>G was significantly associated with EN (*P* = 0.025), birds with the AG genotype had higher EN values. SNP g. 22178975G>A genotypes in* INHA* gene was significantly associated with EN (*P* < 0.05), with genotype GA had more EN than AA genotype (*P *< 0.01). Conversely, chickens with genotype AA had heavier FEW than those with GA genotype.

### 3.3. Construction of Haplotypes and Their Associations with Chicken Reproductive Traits

**Figure 2:**
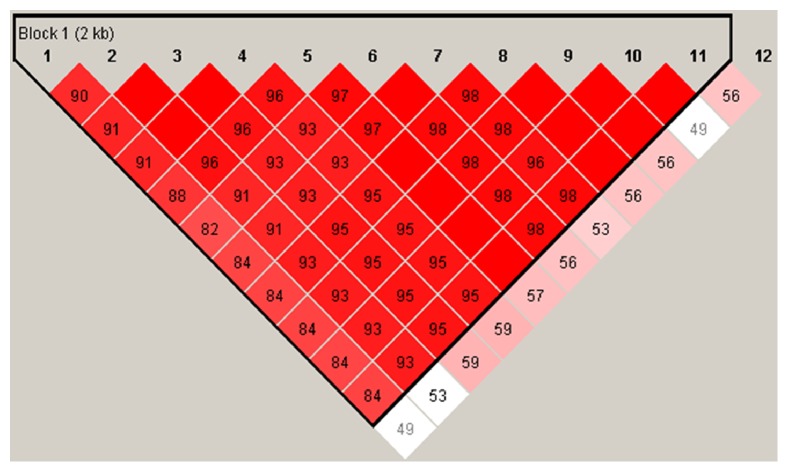
LD analyses of SNPs in the* INHA* gene, the strong LD block is defined as D＇ ≥ 0.8.

Analysis of LD between SNPs in the* INHA* gene was shown in Figure 2. Haplotypes were constructed based on the 12 SNPs identified in LH chickens using the Haploview program (Table 7). H1 was the most frequent haplotype at 53.7 %. Ten diplotypes were obtained based on these five haplotypes (Table 8). However, no significant associations were determined between the reproductive traits in the chicken population and the haplotypes according to regression coefficient analysis.

### 3.4. Expression of INHA in the Chicken Reproductive System

**Figure 3:**
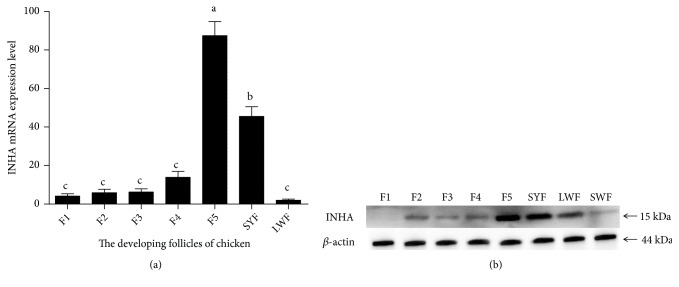
The mRNA and protein expression levels of INHA in chicken reproductive system. (a) The abundance of* INHA* mRNA. (b) INHA protein detected by Western blotting from the chicken preovulatory follicles and normalized to *β*-actin protein content. SWF = small white follicle, LWF = large white follicle, SYF = small yellow follicle, F5 = the fifth largest preovulatory follicle, F4 = the fourth largest preovulatory follicle, F3 = the third largest preovulatory follicle, F2 = the second largest preovulatory follicle, and F1 = the largest preovulatory follicle. Results are expressed as mean ± standard deviation (n = 5). Least square means with different letters differed significantly (*P* < 0.05).

According to expression analysis,* INHA* is mainly expressed in the ovary and follicles (F1-F5, SYF, and LWF). During development of chicken follicles, the relative abundance of* INHA* mRNA initially increased and then decreased, with significantly higher expression in F5 than others (*P *< 0.05). In pre-grade follicles, the* INHA* mRNA expression increased abruptly in SWF follicle (*P *< 0.05). Expression levels in the F1, F2, and F3 follicles were low and did not significantly differ from each other (*P *> 0.05) (Figure 3(a)).

The protein expression levels of INHA in different chicken period follicles were detected by Western blotting, with *β*-actin protein used as a reference. The results showed higher expression of the INHA protein in F5 compared with other follicles (*P *< 0.01) (Figure 3(b)). In the prehierarchical follicles, INHA protein was highly expressed in SYF. These results suggested that* INHA *is a potential candidate gene for improving chicken reproductive traits.

## 4. Discussion

Inhibins play key roles in folliculogenesis, oocyte maturation, and embryo development [26]. The* INHA* gene encodes the functional center of inhibin and may exhibit potential for increasing the ovulation rate in poultry. For example, the downregulation of* INHA* gene expression in cultured goose granulosa cells resulted in significant increases in apoptosis and proliferation indexes, a reduced percentage of cells in the G1 phase, and a correspondingly elevated percentage of cells in the S phase [20]. Moreover, transgenic mice overexpressing the rat* INHA* gene exhibited a reduced litter size and longer intervals between pregnancies when compared with control mice [27]. In some studies, polymorphisms in the* INHA* gene have been found to be significantly associated with follicular cysts in humans [28], pigs [19, 29], and other mammals [30–32]. However,* INHA* polymorphisms associated with reproductive traits in chicken were previously unknown. The purpose of this experiment was to improve the reproductive performance of LH chickens and provide molecular markers for the selective breeding of laying hens.

Ovulation rate is an important reproductive trait, and the important function of* INHA* makes it a strong candidate for improving the poultry ovulation rate. We hypothesized that* INHA* may be a major gene affecting chicken egg production. The AFE, as well as other egg production and egg weight parameters, are important traits used in the breeding of high-quality layers. Therefore, understanding the effects of the* INHA *gene on these reproductive traits is essential. However, information previously available in the literature regarding the relationships between* INHA* polymorphisms and reproductive traits was inconclusive.

Recently, several studies in poultry have been identified crucial genes and explored their relationship with phenotypes, such as Liu with 279 Dongxiang blue-shelled chickens [33], Yu with 188 female Muchuan black-boned chickens [34], and EI-Sabrout with 200 Lohmann Brown hens [35]. In this study, 12 SNPs were detected in* INHA* gene. All SNPs except for SNP1 were in Hardy-Weinberg equilibrium (*P* > 0.05), and their genotype and allele frequencies were above 5 %, which indicate that the population is not affected by artificial selection and its sample size is appropriate for association analysis [36].

Five SNPs (g. 22176870T>C, g. 22177100T>C, g. 22177149T>C, g. 22177991A>G, and g. 22178975G>A) that were significantly associated with chicken EN. This result is similar to that studied in Boer goats, in which the c.651G>A mutation in the* INHA* gene affected the lambing rate [31]. Egg production is an important economic indicator in the poultry industry, and in poultry breeding programs, EN is considered the most valuable indicator of total egg production potential. Our findings therefore confirm the influence of* INHA* polymorphisms on the female reproductive traits of LH chickens. We therefore deduce that* INHA* is an important gene for improving the EN in chickens.

The SNP g. 22177991A>G results in the amino acid substitutions Val→Ala. Zi et al. found that sequence variation in* INHA* was associated with the prolificacy of goat breeds, suggesting that amino acid substitutions in this gene may affect reproductive traits in chicken [37]. While SNP3 results in a synonymous mutation that causes no amino acid change, this mutation was still associated with a reproductive trait. One explanation for this is that this mutation may affect* INHA* function by altering the stability of the mRNA and its translation efficiency. Therefore, this SNP may be associated with the mRNA expression level and increase in* INHA* concentration may lead to decreases in FSH concentrations [38].

Zhu et al. analyzed miRNA-related SNPs and found that two SNPs in the 3＇ UTRs of target genes were predicted to either disturb or create miRNA-target interactions [39]. In the present study, SNP g. 22178618T>C in the 3＇ UTR of* INHA* was predicted to disturb miR-128-3p combination. Yu et al. confirmed the suppression of* CYP2C9* by the miRNA hsa-miR-128-3p in human liver cells and its association with hepatocellular carcinoma [40], while miR-128-3p was found to suppress hepatocellular carcinoma proliferation [41]. SNP g. 22178728C>T was predicted to create bindings sites for two miRNAs miR-125b-5p and miR-34b-5p. miR-125b-5p serves as a novel biomarker for HBV-positive hepatocellular carcinoma [42], and miR-34b-5p inhibits the expression of Bcl-2 in ovarian cancer cells [43]. This suggests that miRNA-related SNPs in the 3＇ UTR of* INHA* may affect poultry reproductive performance. Further studies are needed to confirm that miRNA-related SNPs regulate mRNA and protein expression levels.

Good laying performance of poultry depends on the growth and development of follicles in the ovary. In poultry, only a few of the large number of follicles mature, with only about 5 % developing into SYF. Improving the laying rate and reproductive performance of native chicken breeds has become an urgent concern for chicken breeding and production. The expression levels and forms of inhibin are closely related to physiological activities such as follicular development, recruitment, and dominance selection. Furthermore, as* INHA *is the functional center of inhibin, we hypothesis that the expression level of the chicken* INHA* gene plays a dominant role in follicle development and is related to chicken reproductive traits. Although many studies have shown* INHA* to be a critical regulator of gonadal function, little is known about its expression in chicken developing follicles and the association between its expression and reproductive traits. In this study, we found that the* INHA* gene only expressed in the developing follicles. Intriguingly,* INHA* mRNA expression was the lowest in F1 and highest in F5, consistent with previous results in humans [44]. In the prehierarchical follicles,* INHA* mRNA expression increased sharply in the SWF. Zi et al. previously found that the mRNA expression levels of* INHA* affected prolificacy in goats [37]. The development of LWF into SYF is an important process in the recruitment of follicles in poultry [45]. Additionally, the INHA protein expression profile in the follicle was similar to that of its mRNA counterpart with greater expression in F5 and SYF follicles. These results indicate that the expression of* INHA* is related to follicle development. It can therefore hypothesize that* INHA* is involved in the regulation of follicle development, playing a critical role in follicle recruitment. Further study is needed to illuminate the specific action mechanism of* INHA*.

## 5. Conclusion

Generally, 12 SNPs were identified in chicken* INHA *gene. Five of them were significant associated with egg numbers. Among the exonic SNPs, g. 22177991A>G, g. 22178249G>C, and g. 22178414G>A were missense mutations, which resulted in the amino acid substitutions Val→Ala, Ala→Gly, and Ala→Gly. In addition,* INHA* highly expressed in F5 and SYF follicles. Therefore, we conclude that* INHA* is a candidate gene affecting egg production, and it plays a critical role in the recruitment of follicles in chickens.* INHA* SNPs are possible molecular markers for the genetic selection of layers.

## Figures and Tables

**Table 1:** Primer pairs used to screen the *INHA* gene polymorphism.

Name	Sequences (5＇-3＇)	Binding regions	Product size (bp)	Tm (°C)
P1	F-TATTCACGGCGAGCAGACA	Exon 1	562	58.7
	R-CCTCAGCCCTCCCCATCT			
P2	F-ATCCCACAGCCCCAAGACC	Exon 2	783	61.0
	R-GGCAGTAGTGGAAAACGAAGC			
P3	F-CACTGGACCGTGTTTGACTTCG	Exon 2 Partial	589	51.5
	R-GGGATGGGCTCATCATCTGG			
P4	F-CGGGAACTGTGCCGAAGG	5'UTR	749	58.7
	R-GCACCCCGCTGCATAACC			
P5	F-TGTCCCAAACTCTGTCCAATG	3'UTR	707	56.5
	R-CTCAAATGCTCCAGCACCC			

UTR: untranslated region.

**Table 2:** Summary of variations in the chicken *INHA *gene.

Primer pairs no.	Variations	Chr. position	Gene region	Function
P1	g. 22176805 C>T	22176805	Promoter region	-
P1	g. 22176870T>C	22176870	Exon 1	Synonymous
P2	g. 22177100T>C	22177100	Exon 1	Synonymous
P2	g. 22177149T>C	22177149	Exon 1	Synonymous
P3	g. 22177991A>G	22177991	Exon 2	Missense
P3	g. 22178249G>C	22178249	Exon 2	Missense
P4	g. 22178414G>A	22178414	Exon 2	Missense
P4	g. 22178618T>C	22178618	3＇ UTR	-
P5	g. 22178681A>T	22178681	3＇ UTR	-
P5	g. 22178728C>T	22178728	3＇ UTR	-
P5	g. 22178835A>G	22178835	3＇ UTR	-
P5	g. 22178975G>A	22178975	3＇ UTR	-

UTR: Untranslated region. N =260.

**Table 3:** SNPs in 3＇ UTR of *INHA* gene disturbed or created the binding sites of miRNAs.

Variations	Disturbed miRNAs	Created miRNAs	Binding area
g. 22178618T>C	miR-128-3p	-	22178613-22178619
	miR-6630-3p		22178616-22178621

g. 22178728C>T	-	miR-125b-5p	22178727-22178733
		miR-34b-5p	22178723-22178728
		miR-6673-3p	22178727-22178732

g. 22178835A>G	miR-17-3p	-	22178831-22178837
	miR-6647-5p		22178834-22178839
	miR-6669-3p		22178830-22178835
	miR-7456-5p		22178830-22178837
	miR-7482-3p		22178831-22178837

g. 22178975G>A	miR-181b-1-3p	-	22178972-22178978
	miR-6613-3p		22178972-22178978
	miR-6677-5p		22178973-22178978
	-	miR-460b-5p	22178974-22178980

**Table 4:** Genotypic and allele frequencies and the genetic information of SNP sites of the chicken *INHA* gene.

SNPs	Genotype frequency (%)			Allele frequency (%)		PIC	P-value
SNP1	CC	CT	TT	C	T		
C22176805 T	31.03	61.21	7.76	61.635	38.365	0.3611	P=0.0068
SNP2	TT	TC	CC	T	C		
T22176870C	35.35	55.17	9.48	62.935	37.065	0.3577	P=0.0821
SNP3	TT	TC	CC	T	C		
T22177100C	37.07	54.31	8.62	64.225	35.775	0.3539	P=0.0835
SNP4	TT	TC	CC	T	C		
T22177149C	34.49	56.03	9.48	62.505	37.495	0.3589	P=0.0835
SNP5	AA	AG	GG	A	G		
A22177991G	10.34	52.59	37.07	36.635	63.365	0.3565	P=0.235
SNP6	GG	GC	CC	G	C		
G22178249C	13.80	55.17	31.03	41.385	58.615	0.3675	P=0.2132
SNP7	GG	GA	AA	G	A		
G22178414A	30.17	56.03	13.80	58.185	41.815	0.3682	P=0.1611
SNP8	TT	TC	CC	T	C		
T22178618C	31.03	55.17	13.80	58.615	41.385	0.3675	P=0.2132
SNP9	AA	AT	TT	A	T		
A22178681T	31.03	55.17	13.80	58.615	41.385	0.3675	P=0.2132
SNP10	CC	CT	TT	C	T		
C22178728T	14.66	55.17	30.17	42.245	57.755	0.3689	P=0.2403
SNP11	AA	AG	GG	A	G		
A22178835G	12.94	56.03	31.03	40.955	59.045	0.3667	P=0.2132
SNP12	GG	GA	AA	G	A		
G22178975A	19.83	57.76	22.41	48.71	51.29	0.3748	P=0.1467

P-value is the result of *χ*^2^ test of Hardy-Weinberg equilibrium; PIC < 0.25 indicated low polymorphism, 0.25 < PIC < 0.50 indicated intermediate polymorphism, and PIC > 0.50 indicated high polymorphism.

**Table 5:** Association of *INHA* polymorphisms with chicken reproductive traits.

Polymorphism	Traits (P value of significant test)					
	AFE(days)	BWFE(g)	FEW(g)	BWTA(g)	EWTA(g)	EN(count)
g. 22176805 C>T	0.838	0.407	0.962	0.308	0.192	0.353
g. 22176870T>C	0.214	0.406	0.998	0.457	0.876	0.017*∗*
g. 22177100T>C	0.296	0.259	0.954	0.222	0.846	0.022*∗*
g. 22177149T>C	0.516	0.203	0.967	0.211	0.820	0.033*∗*
g. 22177991A>G	0.279	0.179	0.990	0.321	0.768	0.025*∗*
g. 22178249G>C	0.569	0.240	0.812	0.504	0.384	0.150
g. 22178414G>A	0.707	0.393	0.757	0.618	0.380	0.259
g. 22178618T>C	0.569	0.185	0.830	0.344	0.573	0.124
g. 22178681A>T	0.569	0.185	0.830	0.344	0.573	0.124
g. 22178728C>T	0.628	0.242	0.859	0.452	0.416	0.117
g. 22178835A>G	0.569	0.185	0.830	0.344	0.573	0.124
g. 22178975G>A	0.282	0.126	0.106	0.133	0.117	0.018*∗*

*∗ P *≤ 0.05; AFE=age at first egg; BWFE=body weight at first egg; FEW= first egg weight; BWTA=body weight at 300 days of age; EWTA=egg weight at 300 days of age; EN=total number of eggs at 300 days of age.

**Table 6:** Association analyses between SNPs of chicken *INHA* gene and EN.

SNP	Genotypes			P-value
g. 22176870T>C	TT	TC	CC	
	111.512±1.928^b^	117.516±1.544^a^	109.000±3.723^b^	0.017
g. 22177100T>C	TT	TC	CC	
	111.927±1.939^b^	117.250±1.552^a^	109.000±3.744^b^	0.022
g. 22177149T>C	TT	TC	CC	
	112.070±1.887^b^	117.365±1.559^a^	107.900±3.913^b^	0.033
g. 22177991A>G	AA	AG	GG	
	111.083±3.576^b^	117.607±1.586^a^	111.279±1.889^b^	0.025
g. 22178975G>A	GG	GA	AA	
	108.769±2.422^b^	114.130±2.575^b^	117.000±1.509^a^	0.018

Results are expressed as mean ± standard errors. Values in the table were the total number of eggs at 300 days of age for different genotypes. Different letters indicate significant differences (*P *< 0.05).

**Table 7:** Haplotypes inferred based on the twelve SNPs.

Haplotype	SNP1	SNP2	SNP3	SNP4	SNP5	SNP6	SNP7	SNP8	SNP9	SNP10	SNP11	Frequency
H1	C	T	T	T	G	C	G	T	A	T	G	0.54
H2	T	C	C	C	A	G	A	C	T	C	A	0.33
H3	C	T	T	T	G	G	A	C	T	C	A	0.05
H4	T	T	T	T	G	C	G	T	A	T	G	0.02
H5	C	C	C	C	A	G	A	C	T	C	A	0.02

**Table 8:** Diplotypes of chicken* INHA* gene.

Diplotypes	H1H1	H1H2	H1H3	H1H4	H2H2	H2H3	H2H4	H2H5	H3H4	H4H4
Frequency (%)	24.14	47.41	4.3	0.86	5.17	3.45	0.86	3.45	0.86	0.86

## Data Availability

The data used to support the findings of this study are available from the corresponding author upon request.
